# The tigecycline evaluation and surveillance trial; assessment of the activity of tigecycline and other selected antibiotics against gram-positive and gram-negative pathogens from France collected between 2004 and 2016

**DOI:** 10.1186/s13756-018-0360-y

**Published:** 2018-05-30

**Authors:** Jean-Winoc Decousser, Paul-Louis Woerther, Claude-James Soussy, Marguerite Fines-Guyon, Michael J. Dowzicky

**Affiliations:** 1 University Hospital Henri Mondor, 9400 Creteil, France; 2 Caen University Hospital, 14033 Caen, Cedex 9, France; 3 Pfizer Inc, Collegeville, PA USA

**Keywords:** France, Gram-positive, Gram-negative, Multidrug-resistance, Antimicrobial surveillance, Tigecycline

## Abstract

**Background:**

A high level of antibiotic consumption in France means antimicrobial resistance requires rigorous monitoring. The Tigecycline Evaluation and Surveillance Trial (T.E.S.T.) is a global surveillance study that monitors the in vitro activities of tigecycline and a panel of marketed antimicrobials against clinically important Gram-positive and Gram-negative isolates.

**Methods:**

Annually clinically relevant strains were prospectively included in the survey through a national network of hospital-based laboratories. MICs were determined locally by broth microdilution using CLSI guidelines. Antimicrobial susceptibility was assessed using European Committee on Antimicrobial Susceptibility Testing breakpoints.

**Results:**

Thirty-three centres in France collected 26,486 isolates between 2004 and 2016. *Enterococcus* species were highly susceptible (≥94.4%) to linezolid, tigecycline and vancomycin. *Staphylococcus aureus*, including methicillin-resistant *S. aureus* (MRSA), were susceptible (≥99.9%) to tigecycline, vancomycin and linezolid. Between 2004 and 2016, 27.7% of *S. aureus* isolates were MRSA, decreasing from 28.0% in 2013 to 23.5% in 2016. Susceptibility of *Streptococcus pneumoniae* isolates was 100% to vancomycin, and > 99.0% to levofloxacin, linezolid and meropenem; 3.0% were penicillin-resistant *S. pneumoniae* (100% susceptibility to vancomycin and linezolid). *Escherichia coli* isolates were highly susceptible (> 98.0%) to meropenem, tigecycline and amikacin. The rate of extended-spectrum β-lactamase (ESBL) positive *E. coli* increased from 2004 (3.0%), but was stable from 2012 (23.1%) to 2016 (19.8%). Susceptibility of *Klebsiella pneumoniae* isolates was 99.4% to meropenem and 96.5% to amikacin. The proportion of ESBL-positive *K. pneumoniae* isolates increased from 2004 (7.5%) to 2012 (33.3%) and was highest in 2016 (43.6%). *A. baumannii* was susceptible to meropenem (81.0%) and amikacin (74.9%); none of the 6.2% of isolates identified as multidrug-resistant (MDR) was susceptible to any agents with breakpoints. *P. aeruginosa* isolates were most susceptible to amikacin (88.5%), and MDR rates were 13.6% in 2013 to 4.0% in 2016; susceptibility of MDR isolates was no higher than 31.4% to amikacin.

**Conclusions:**

Rates of MRSA decreased slowly, while rates of ESBL-positive *E. coli* and *K. pneumoniae* increased from 2004 to 2016. Susceptibility of Gram-positive isolates to vancomycin, tigecycline, meropenem and linezolid was well conserved, as was susceptibility of Gram-negative isolates to tigecycline and meropenem. The spread of MDR non-fermentative isolates must be carefully monitored.

## Background

Despite significant efforts to reduce antibiotic use, France has one of the highest rates of antimicrobial consumption in the community in Europe [1], and has seen considerable changes in trends of antibacterial resistance during recent years [2–5]. In France, resistance to antibiotics has been monitored since 2002 by the French national healthcare-associated infection early warning, investigation and surveillance network (RAISIN), which recently reported a 182% increase in the prevalence of extended-spectrum β-lactamase (ESBL)-producing Enterobacteriaceae during nine years [2]. Extensively drug-resistant bacteria such as vancomycin-resistant enterococci (VRE) and carbapenemase-producing Enterobacteriaceae (CPE) are not endemic in France, although VRE are disseminated in neighbouring countries such as Italy and Germany, and CPE are considered endemic in Italy [6, 7]. Methicillin-resistant *Staphylococcus aureus* (MRSA) rates in France have been considered to be decreasing during the decade from 2000 to 2010 and in subsequent years [3, 8, 9], and this is consistent with reduced MRSA rates reported in Germany since 2007 [3, 10–12] and from 2010 in the UK [3, 13]. The situation regarding antimicrobial resistance in France requires rigorous monitoring, particularly for second-line antimicrobial compounds and clinically relevant bacterial species. To meet the challenge presented by antimicrobial resistance, authorities in France have developed a number of national initiatives that include antibiotic stewardship in hospitals and surveillance of antibiotic use [14].

The broad-spectrum antimicrobial agent tigecycline is indicated for the treatment of complicated skin and soft tissue infections (cSSTIs), excluding diabetic foot infections, and complicated intra-abdominal infections (cIAIs), and, in the USA, community-acquired bacterial pneumonia [15, 16]. The Tigecycline Evaluation and Surveillance Trial (T.E.S.T.) was instigated in 2004 with the intention of global surveillance of antimicrobial activity of tigecycline and a panel of other antimicrobial agents against an array of clinically important Gram-positive and Gram-negative pathogens. In this study, we report an update to that provided by Cattoir and Dowzicky [17] regarding the in vitro susceptibility to tigecycline and comparators of isolates collected from community or hospitalized patients in France between 2004 and 2016.

## Methods

Materials and methods for isolates collected as part of the T.E.S.T. study in France have been published previously [17], with minimum inhibitory concentrations (MICs) determined locally according to the broth microdilution method described by the Clinical and Laboratory Standards Institute (CLSI) [18, 19].

Isolates were collected if considered to be of clinical significance as the probable causative agent of a hospital- or community-acquired infection. Isolates were accepted from all body sites, including the following sources: samples of body fluids (classified as abdominal, ascites, bile, paracentesis, peritoneal), central nervous system, cardiovascular system, gastrointestinal (GI) sources (abscess, appendix, diverticulum, oesophagus, faeces/stool, gall bladder, large colon, liver, pancreas, rectum, small colon, stomach, general GI or other GI), genito-urinary, head, ears, eyes, nose and throat, integument, lymph, muscular, reproductive, respiratory, skeletal or medical instruments (i.e. catheters, drains, forceps, probes). Duplicate isolates from a single patient were not accepted.

Coordination of isolate collection and transport was carried out by International Health Management Associates (IHMA), Schaumburg, IL, USA. The panel of antimicrobial agents for the T.E.S.T. study included an aminoglycoside (amikacin), agents in the penicillin class (ampicillin, amoxicillin-clavulanate, penicillin, piperacillin-tazobactam), cephalosporins (cefepime, ceftazidime, ceftriaxone) a carbapenem (imipenem), a fluoroquinolone (levofloxacin), an oxazolidinone (linezolid), a tetracycline (minocycline), a glycylcycline (tigecycline) and a glycopeptide (vancomycin). In 2006, meropenem replaced imipenem due to stability issues associated with imipenem testing, and the *S. pneumoniae* test panel was expanded to include three macrolides (azithromycin, clarithromycin, erythromycin) and a lincosamide (clindamycin), with isolates tested retrospectively for susceptibility to these agents wherever possible. Antimicrobial susceptibility of aerobic isolates was performed using the breakpoints established by the European Committee on Antimicrobial Susceptibility Testing (EUCAST) [20]. Susceptibility data are included in the tables only when interpretive breakpoints are available. Methicillin resistance in *S. aureus* and ESBL-production among *E. coli* and *Klebsiella* spp. were determined by IHMA according to CLSI guidelines [19]. As specified in a previous T.E.S.T. study [21], isolates that were resistant to three or more classes of antimicrobial agents were defined as multidrug-resistant (MDR). Classes used to define MDR *A. baumannii* were aminoglycosides (amikacin), β-lactams (cefepime, ceftazidime, ceftriaxone or piperacillin-tazobactam), carbapenems (imipenem/meropenem), fluoroquinolones (levofloxacin) and tetracyclines (minocycline), and the classes used to define MDR *P. aeruginosa* were aminoglycosides (amikacin), β-lactams (cefepime, ceftazidime, or piperacillin-tazobactam), carbapenems (imipenem/meropenem), and fluoroquinolones (levofloxacin) [21].

The Cochran Armitage Trend Test was used to identify statistically significant changes in susceptibility between 2004 and 2016, and results with a *p*-value of < 0.01 were deemed significant.

## Results

A total of 26,486 isolates were collected from 33 centres in France between 2004 and 2016 (eight in 2004, six in 2005, 12 in 2006, 16 in 2007, 21 in 2008, 20 in 2009, 16 in 2010 and 2011, 14 in 2012, 12 in 2013 and 2014, 11 in 2015 and four in 2016).

### Gram-positives

#### Enterococcus spp

All isolates of *E. faecalis* (*N* = 1429) were highly susceptible (≥98.4%) to ampicillin, linezolid, tigecycline and vancomycin (Table 1). All isolates of VRE *E. faecalis* (*N* = 11, 0.8%) were susceptible to tigecycline and 90.9% were susceptible to linezolid. Between 2004 and 2016, 537 isolates of *E. faecium* were collected, which included 410 (76.4%) ampicillin-resistant isolates. All isolates were highly susceptible to tigecycline (100%), linezolid (99.8%) and vancomycin (94.4%) (Table 1). Thirty *E. faecium* isolates (5.6%) were identified as VRE, which were 100% susceptible to linezolid and tigecycline.

**Table 1:** Minimum inhibitory concentrations (MIC_90_, MIC range [mg/L]) and antimicrobial susceptibility (%S) and resistance (%R) of Gram-positive isolates

Organism/ Antimicrobial	2004–2016				2013–2016			
	MIC_90_ (mg/L)	MIC Range (mg/L)	% S	% R	MIC_90_ (mg/L)	MIC Range (mg/L)	% S	% R
*E. faecalis*	*N* = 1429				*N* = 373			
Ampicillin^a^	2	≤0.06 to ≥32	98.4	1.0	1	≤0.06 to ≥32	97.1	2.1
Linezolid	2	≤0.5 to ≥16	99.9	0.1	2	≤0.5 to ≥16	99.7	0.3
Tigecycline	0.25	≤0.008 to 0.5	99.9	0.0	0.12	0.03 to 0.25	100	0.0
Vancomycin	2	0.25 to ≥64	99.2	0.8	2	0.25 to ≥64	99.2	0.8
*E. faecalis,* VRE	*N* = 11				*N* = 3			
Amox-clav	≥16	0.25 to ≥16	81.8	18.2	≥16	0.5 to ≥16	[1]	[2]
Ampicillin	≥32	0.5 to ≥32	81.8	18.2	≥32	1 to ≥32	[1]	[2]
Linezolid	2	1 to ≥16	90.9	9.1	≥16	1 to ≥16	[2]	[1]
Tigecycline	0.25	0.06 to 0.25	100	0.0	0.25	0.06 to 0.25	[3]	[0]
*E. faecium*	*N* = 537				*N* = 159			
Linezolid	2	≤0.5 to 8	99.8	0.2	2	≤0.5 to 8	99.4	0.6
Tigecycline	0.25	0.015 to 0.25	100	0.0	0.12	0.015 to 0.25	100	0.0
Vancomycin	2	0.25 to ≥64	94.4	5.6	1	0.25 to ≥64	98.1	1.9
*E. faecium*, VRE	*N* = 30				*N* = 3			
Linezolid	2	1 to 2	100	0.0	2	1 to 2	[3]	[0]
Tigecycline	0.25	0.03 to 0.25	100	0.0	0.25	0.06 to 0.25	[3]	[0]
*S. aureus*	*N* = 3437				*N* = 947			
Levofloxacin^b^	32	≤0.06 to ≥64	73.2	26.8	16	≤0.06 to ≥64	76.7	23.3
Linezolid	2	≤0.5 to 8	> 99.9	< 0.1	2	≤0.5 to 8	99.9	0.1
Minocycline^b^	0.5	≤0.25 to ≥16	95.0	3.0	≤0.25	≤0.25 to ≥16	97.5	2.1
Penicillin	≥16	≤0.06 to ≥16	15.0	85	≥16	≤0.06 to ≥16	16.6	83.4
Tigecycline	0.25	≤0.008 to 0.5	100	0.0	0.12	0.015 to 0.5	100	0.0
Vancomycin	1	≤0.12 to 2	100	0.0	1	0.25 to 2	100	0.0
*S. aureus*, MRSA	*N* = 953				*N* = 234			
Levofloxacin^b^	≥64	≤0.06 to ≥64	16.7	83.3	32	0.12 to ≥64	17.5	82.5
Linezolid	2	≤0.5 to 4	100	0.0	4	≤0.5 to 4	100	0.0
Minocycline^b^	0.5	≤0.25 to ≥16	94.2	4.6	≤0.25	≤0.25 to 8	95.3	3.8
Penicillin	≥16	0.5 to ≥16	0.0	100	≥16	0.25 to ≥16	0.0	100
Tigecycline	0.25	0.015 to 0.25	100	0.0	0.25	0.015 to 0.5	100	0.0
Vancomycin	1	≤0.12 to 2	100	0.0	1	0.25 to 2	100	0.0
*S. agalactiae*	*N* = 1348				*N* = 378			
Levofloxacin	1	≤0.06 to 32	99.1	0.9	1	0.12 to 32	97.9	2.1
Linezolid	1	≤0.5 to 2	100	0.0	1	≤0.5 to 2	100	0.0
Minocycline	≥16	≤0.25 to ≥16	16.1	82.0	≥16	≤0.25 to ≥16	15.6	83.1
Penicillin	0.12	≤0.06 to 0.12	100	0.0	0.12	≤0.06 to 0.12	100	0.0
Tigecycline	0.12	0.015 to 4	99.8	0.1	0.12	0.015 to 4	99.7	0.3
Vancomycin	0.5	≤0.12 to 1	100	0.0	0.5	≤0.12 to 1	100	0.0
*S. pneumoniae*	*N* = 1684 (AZM, CLR, CLI, ERY, *N* = 1500)				*N* = 496 (AZM, CLR, CLI, ERY, N = 473)			
Azithromycin^b^	≥128	≤0.03 to ≥128	60.5	39.1	≥128	≤0.03 to ≥128	65.8	33.8
Ceftriaxone	1	≤0.03 to 16	80.8	0.5	1	≤0.03 to 2	84.7	0.0
Clarithromycin^b^	≥128	≤0.015 to ≥128	60.9	38.5	≥128	≤0.015 to ≥128	66.4	32.6
Clindamycin^b^	≥128	≤0.015 to ≥128	68.5	31.5	≥128	≤0.015 to ≥128	71.9	28.1
Erythromycin^b^	≥128	≤0.015 to ≥128	60.6	38.7	≥128	≤0.015 to ≥128	66.4	33.2
Levofloxacin	1	≤0.06 to ≥64	99.7	0.3	1	≤0.06 to 2	100	0.0
Linezolid	1	≤0.5 to 4	99.9	0.0	1	≤0.5 to 2	100	0.0
Meropenem (N = 1557) ^c^	0.5	≤0.12 to ≥32	99.8	0.2	1	≤0.12 to 8	99.8	0.2
Minocycline^b^ (*N* = 1683)	8	≤0.25 to ≥16	52.4	38.6	8	≤0.25 to ≥16	61.7	31.9
Penicillin	2	≤0.06 to ≥16	53.0	3.0	2	≤0.06 to 8	53.4	2.8
Tigecycline	0.06	≤0.008 to 0.5	–	–	0.03	≤0.008 to 0.06	–	–
Vancomycin	0.5	≤0.12 to 1	100	0.0	0.5	≤0.12 to 1	100	0.0
*S. pneumoniae,* PRSP	*N* = 51 (AZM, CLR, CLI, ERY, N = 48)				*N* = 14 (AZM, CLR, CLI, ERY, N = 14)			
Azithromycin	≥128	≤0.03 to ≥128	22.9	77.1	≥128	0.06 to ≥128	38.5	61.5
Ceftriaxone	2	≤0.03 to 8	9.8	9.8	2	≤0.03 to 2	21.4	0
Clarithromycin	≥128	≤0.015 to ≥128	22.9	77.1	≥128	≤0.015 to ≥128	38.5	61.5
Clindamycin	≥128	≤0.015 to ≥128	37.5	62.5	≥128	0.03 to ≥128	46.2	53.8
Erythromycin	≥128	≤0.015 to ≥128	22.9	75.0	≥128	0.03 to ≥128	38.5	61.5
Levofloxacin	1	0.25 to 16	98.0	2.0	1	0.5 to 1	100	0.0
Linezolid	1	≤0.5 to 2	100	0.0	1	≤0.5 to 2	100	0.0
Meropenem^c^	2	≤0.12 to ≥32	94.1	5.9	2	≤0.12 to 8	92.9	7.1
Minocycline	≥16	≤0.25 to ≥16	19.6	70.6	8	≤0.25 to ≥16	21.4	64.3
Tigecycline	0.03	0.015 to 0.12	–	–	0.03	0.015 to 0.03	–	–
Vancomycin	0.5	0.25 to 1	100	0.0	0.5	025 to 1	100	0.0

^a^ indicates statistically significant decrease in susceptibility (*p* < 0.01) from 2004 to 2016

^b^ indicates statistically significant increase in susceptibility (*p* < 0.01) from 2004 to 2016

^c^ Meropenem was introduced to the testing panel in 2006, replacing imipenem; N values of activity against organisms collected from 2006 to 2016 are given

*Amox-clav*, amoxicillin-clavulanic acid, *AZM*, azithromycin, *CLR*, clarithromycin, *CLI*, clindamycin, *ERY*, erythromycin, *MIC*, minimum inhibitory concentration, *MIC*_*90*_, minimum inhibitory concentration required to inhibit growth of 90% of isolates (mg/L), *MRSA*, methicillin-resistant *S. aureus*, *Pip-taz*, piperacillin-tazobactam, *PRSP*, Penicillin-resistant *S. pneumoniae*, *R*, resistant, *S*, susceptible, *VRE*, vancomycin-resistant enterococci

#### S. Aureus

All *S. aureus* isolates (*N* = 3437) were susceptible to tigecycline and vancomycin (Table 1). Susceptibility to linezolid was > 99.9%, to minocycline 95.0% and to levofloxacin 73.2%. The proportion of isolates identified as MRSA (*N* = 953) between 2004 to 2016 was 27.7% (range, 18.3–34.3%) and during the period 2013 to 2016 decreased from 28.0 to 23.5% (Table 2). All MRSA isolates were susceptible to linezolid, tigecycline and vancomycin (Table 1), and susceptibility to minocycline was 94.2%. The susceptibility of MRSA isolates collected between 2004 and 2016 to levofloxacin was relatively low, at 16.7%. A vancomycin MIC of > 1 mg/L was observed in 35 (3.7%) of the MRSA isolates, and of these, 2.9% were susceptible to levofloxacin, and 74.3% to minocycline. MRSA isolates that exhibited a vancomycin MIC that was ≤1 mg/L (*N* = 918) exhibited susceptibility of 17.2% to levofloxacin and 95.0% to minocycline.

**Table 2:** Percentages of resistant phenotypes among Gram-positive and Gram-negative isolates by year, 2013—2016

	*E. coli* ESBL-positive		*K. pneumoniae* ESBL-positive		*H. influenzae* BL positive		*P. aeruginosa* MDR		*A. baumannii* MDR		MRSA	
	n	%	n	%	n	%	n	%	n	%	n	%
2013	46	14.9	75	36.1	39	25.3	33	13.6	11	12.2	84	28.0
2014	43	15.6	85	40.7	27	18.9	21	9.8	10	13.5	76	27.9
2015	47	16.8	71	36.4	36	25.7	11	5.3	7	9.1	50	18.3
2016	20	19.8	34	43.6	20	35.1	3	4.0	7	24.1	24	23.5

*BL*, β-lactamase, *ESBL*, extended-spectrum β-lactamase, *MDR*, multidrug-resistant, *MRSA*, methicillin-resistant *S. aureus*

#### S. Agalactiae

Susceptibility of *S. agalactiae* isolates (*N* = 1348) was 100% to linezolid, penicillin and vancomycin; isolates were also highly susceptible to tigecycline (99.8%), and to levofloxacin (99.1%).

#### S. Pneumoniae

A total of 1684 isolates of *S. pneumoniae* were collected during the study, and all were susceptible to vancomycin, with > 99.6% of isolates susceptible to levofloxacin, linezolid and meropenem (*N* = 1557 for meropenem). Tigecycline exhibited an in vitro MIC_90_ value of 0.06 mg/L against *S. pneumoniae* isolates, and during the study there was a statistically significant increase (*p* < 0.0001) in susceptibility to azithromycin (2004, 50.0%; 2016, 76.2%), clarithromycin (2004, 50.0%; 2016, 78.6%), clindamycin (2004, 52.3%; 2016, 83.3%) and erythromycin (2004, 50.0%; 2016, 78.6%), and also to minocycline (*p* < 0.01; 2004, 52.7%; 2016, 78.6%). A total of 51 (3.0%) penicillin-resistant *S. pneumoniae* isolates were collected between 2004 to 2016 and all of these were susceptible to vancomycin and linezolid. Rates of penicillin-resistant *S. pneumoniae* susceptibility to levofloxacin (98.0%) and meropenem (94.1%) were relatively high and stable; the MIC_90_ of tigecycline was 0.03 mg/L. Penicillin-resistant *S. pneumoniae* isolates collected between 2013 and 2016 and tested for susceptibility to erythromycin (*N* = 13) and minocycline (*N* = 14) showed susceptibility rates of 38.5 and 21.4%, respectively, which were lower compared with all *S. pneumoniae* isolates that were collected during the same period and tested against erythromycin (*N* = 473, 66.4% susceptibility) and minocycline (*N* = 496, 61.7% susceptibility).

### Gram-negatives

#### Enterobacter spp

The agent with the lowest in vitro MIC_90_ value against *Enterobacter* spp. isolates (*N* = 3424) was meropenem (MIC_90_ 0.25 mg/L), to which 99.2% of isolates were susceptible (Table 3). Susceptibility to amikacin (96.9%) and tigecycline (86.3%) was stable, and susceptibility to levofloxacin was 71.5%. A lower proportion of isolates were susceptible to the cephalosporins on the T.E.S.T. panel, cefepime (69.5%) and ceftriaxone (50.9%).

**Table 3:** Minimum inhibitory concentrations (MIC_90_, MIC range [mg/L]) and antimicrobial susceptibility (%S) and resistance (%R) of Gram-negative isolates

Organism/ Antimicrobial	2004–2016				2013–2016			
	MIC_90_ (mg/L)	MIC Range (mg/L)	% S	% R	MIC_90_ (mg/L)	MIC Range (mg/L)	% S	% R
*Enterobacter* spp.	*N* = 3424				*N* = 924			
Amikacin	4	≤0.5 to ≥128	96.9	1.1	4	≤0.5 to ≥128	98.4	0.8
Cefepime^a^	16	≤0.5 to ≥64	69.5	15.8	32	≤0.5 to ≥64	67.1	19.8
Ceftriaxone	64	≤0.06 to ≥128	50.9	45.6	64	≤0.06 to 64	49.1	47.3
Levofloxacin^c^	≤16	≤0.008 to ≥16	71.5	25.0	8	≤0.008 to ≥16	75.8	19.7
Meropenem (*N* = 3113)^b^	0.25	≤0.06 to ≥32	99.2	0.3	0.25	≤0.06 to ≥32	99.4	0.2
Minocycline	16	≤0.5 to ≥32	–	–	8	≤0.5 to ≥32	–	–
Pip-taz^c^	128	≤0.06 to ≥256	60.7	30.5	128	≤0.06 to ≥256	64.7	25.6
Tigecycline	2	0.06 to 16	86.3	5.2	2	0.06 to 16	89.5	3.5
*E. coli*	N = 3527				*N* = 965			
Amikacin	4	≤0.5 to ≥128	98.1	0.5	4	≤0.5 to ≥128	98.4	0.2
Amox-clav	32	0.25 to ≥64	72.1	27.9	16	0.5 to ≥64	75.1	24.9
Ampicillin	≥64	≤0.5 to ≥64	37.9	62.1	≥64	≤0.5 to ≥64	38.5	61.5
Cefepime^a^	8	≤0.5 to ≥64	82.5	12.4	16	≤0.5 to ≥64	80.7	13.8
Ceftriaxone^a^	64	≤0.06 to ≥128	82.6	16.8	64	≤0.06 to 64	81.3	18.4
Levofloxacin	≥16	≤0.008 to ≥16	78.5	20.2	8	≤0.008 to ≥16	79.6	19.4
Meropenem (*N* = 3203)^b^	≤0.06	≤0.06 to 8	99.9	0.0	≤0.06	≤0.06 to 8	99.9	0.0
Minocycline	8	≤0.5 to ≥32	–	–	8	≤0.5 to ≥32	–	–
Pip-taz	16	≤0.06 to ≥256	89.6	7.2	8	≤0.06 to ≥256	91.6	6.2
Tigecycline	0.5	≤0.008 to 16	99.4	0.1	0.25	0.03 to 16	99.5	0.1
*E. coli*, ESBL	*N* = 489				*N* = 156			
Amikacin	8	≤0.5 to ≥128	92.6	2.2	8	1 to ≥128	95.5	0.6
Amox-clav^c^	32	2 to ≥64	45.8	54.2	16	2 to ≥64	59.0	41.0
Ampicillin	≥64	32 to ≥64	0.0	100	≥64	64 to ≥64	0.0	100
Cefepime	≥64	≤0.5 to ≥64	3.9	78.3	≥64	1 to ≥64	3.2	79.5
Ceftriaxone	≥128	2 to ≥128	0.0	99.2	64	4 to 64	0.0	100
Levofloxacin	≥16	≤0.008 to ≥16	37.8	59.7	≥16	0.015 to ≥16	42.3	55.1
Meropenem (*N* = 472)^b^	≤0.06	≤0.06 to 2	100	0.0	≤0.06	≤0.06 to 1	100	0.0
Minocycline	16	≤0.5 to ≥32	–	–	16	≤0.5 to ≥32	–	–
Pip-taz^c^	32	0.25 to ≥256	78.3	12.7	16	0.25 to ≥256	88.5	3.8
Tigecycline	0.5	0.03 to 2	99.2	0.0	0.25	0.03 to 2	99.4	0.0
*H. influenzae*	*N* = 1786				*N* = 494			
Amikacin	8	≤0.5 to 64	–	–	8	≤0.5 to 16	–	–
Amox-clav	1	≤0.12 to 16	99.2	0.8	1	≤0.12 to 4	99.0	1.0
Ampicillin	32	≤0.5 to ≥64	75.4	24.6	32	≤0.5 to ≥64	74.1	25.9
Cefepime	≤0.5	≤0.5 to 2	–	–	≤0.5	≤0.5 to 2	–	–
Ceftriaxone	≤0.06	≤0.06 to 4	98.6	1.4	≤0.06	≤0.06 to 2	99.4	0.4
Levofloxacin	0.015	≤0.008 to 8	98.4	1.6	0.015	≤0.008 to 8	98.6	1.4
Meropenem (*N* = 1629)^b^	0.12	≤0.06 to 0.5	100	0.0	0.12	≤0.06 to 0.5	100	0.0
Minocycline	1	≤0.5 to 16	91.8	1.6	1	≤0.5 to 4	93.1	0.8
Pip-taz	≤0.06	≤0.06 to 0.5	–	–	≤0.06	≤0.06 to 0.5	–	–
Tigecycline	0.25	≤0.008 to 4	–	–	0.25	≤0.008 to 0.25	–	–
*H. influenzae,* BL Positive	*N* = 410				*N* = 122			
Amikacin	8	≤0.5 to 32	–	–	8	≤0.5 to 16	–	–
Amox-clav	2	≤0.12 to 16	97.3	2.7	2	≤0.12 to 4	95.9	4.1
Ampicillin	≥64	≤0.5 to ≥64	0.5	99.5	≥64	≤0.5 to ≥64	0.8	99.2
Cefepime	≤0.5	≤0.5 to 2	–	–	≤0.5	≤0.5 to 2	–	–
Ceftriaxone	≤0.06	≤0.06 to 4	97.8	2.2	≤0.06	≤0.06 to 2	99.2	0.8
Levofloxacin	0.03	≤0.008 to 1	97.8	2.2	0.015	≤0.008 to 0.5	97.5	2.5
Meropenem (*N* = 378)^b^	0.12	≤0.06 to 0.5	100	0.0	0.12	≤0.06 to 0.5	100	0.0
Minocycline	1	≤0.5 to 16	93.2	0.5	1	≤0.5 to 2	93.4	0.0
Pip-taz	≤0.06	≤0.06 to 0.5	–	–	≤0.06	≤0.06 to 0.5	–	–
Tigecycline	0.25	≤0.008 to 0.5	–	–	0.25	≤0.008 to 0.25	–	–
*K. oxytoca*	*N* = 975				*N* = 225			
Amikacin	4	≤0.5 to ≥128	98.9	0.4	4	≤0.5 to 16	99.1	0.0
Amox-clav	32	0.25 to ≥64	79.8	20.2	16	0.25 to ≥64	82.2	17.8
Cefepime	2	≤0.5 to ≥64	88.4	3.9	2	≤0.5 to ≥64	88.4	4.9
Ceftriaxone	8	≤0.06 to ≥128	83.2	14.5	4	≤0.06 to 64	85.8	12.0
Levofloxacin	1	≤0.008 to ≥16	89.5	8.4	0.25	0.015 to ≥16	94.2	4.4
Meropenem (*N* = 872)^b^	≤0.06	≤0.06 to ≥32	99.8	0.1	≤0.06	≤0.06 to 1	100	0.0
Minocycline	4	≤0.5 to ≥32	–	–	2	≤0.5 to 16	–	–
Pip-taz	≥256	≤0.06 to ≥256	84.0	15.1	64	0.25 to ≥256	87.6	11.6
Tigecycline	1	0.015 to 8	95.8	1.0	0.5	0.12 to 4	96.9	0.9
*K. pneumoniae*	*N* = 2398				*N* = 690			
Amikacin	4	≤0.5 to ≥128	96.5	1.5	4	≤0.5 to ≥128	96.8	1.7
Amox-clav^a^	32	0.5 to ≥64	68.6	31.4	32	1 to ≥64	61.4	38.6
Cefepime^a^	≥64	≤0.5 to ≥64	72.1	23.4	≥64	≤0.5 to ≥64	59.9	35.5
Ceftriaxone^a^	64	≤0.06 to ≥128	70.3	28.7	64	≤0.06 to 64	58.4	41.4
Levofloxacin^a^	8	≤0.008 to ≥16	76.1	20	8	0.015 to ≥16	72.3	23.2
Meropenem^a^ (*N* = 2186)^b^	0.12	≤0.06 to ≥32	99.4	0.4	0.12	≤0.06 to ≥32	98.8	1.0
Minocycline	16	≤0.5 to ≥32	–	–	16	≤0.5 to ≥32	–	–
Pip-taz	64	0.12 to ≥256	81.9	13.1	32	0.12 to≥256	84.1	10.3
Tigecycline^a^	2	0.06 to 16	87.4	5	2	0.06 to 8	86.2	7.0
*K. pneumoniae,* ESBL	*N* = 622				*N* = 265			
Amikacin	8	≤0.5 to ≥128	90.0	4.2	8	≤0.5 to ≥128	94.7	3.8
Amox-clav	32	1 to ≥64	19.0	81.0	32	1 to ≥64	20.8	79.2
Cefepime^a^	≥64	≤0.5 to ≥64	5.0	85.0	≥64	≤0.5 to ≥64	3.8	86.8
Ceftriaxone	≥128	≤0.06 to ≥128	1.3	98.4	64	≤0.06 to 64	1.1	98.9
Levofloxacin^c^	≥16	0.03 to ≥16	30.2	61.1	≥16	0.03 to ≥16	38.9	50.9
Meropenem (*N* = 603)^b^	0.12	≤0.06 to ≥32	99.0	0.3	0.12	≤0.06 to 16	99.2	0.4
Minocycline	≥32	≤0.5 to ≥32	–	–	≥32	≤0.5 to ≥32	–	–
Pip-taz^c^	≥256	0.25 to ≥256	54.0	32.8	128	0.25 to ≥256	68.7	18.5
Tigecycline	2	0.12 to 8	79.4	7.2	2	0.12 to 8	80.0	7.9
*S. marcescens*	*N* = 1345				*N* = 360			
Amikacin	4	≤0.5 to ≥128	97.3	1.1	4	≤0.5 to 64	98.3	0.6
Cefepime	≤0.5	≤0.5 to ≥64	94.5	2.2	≤0.5	≤0.5 to ≥32	94.7	1.9
Ceftriaxone	8	≤0.06 to ≥128	82.2	13.8	2	≤0.06 to 64	86.9	8.9
Levofloxacin^c^	2	≤0.008 to ≥16	84.2	10.6	1	≤0.008 to ≥16	89.4	5.6
Meropenem (*N* = 1227)^b^	0.12	≤0.06 to ≥32	99.1	0.1	0.12	≤0.06 to 2	100	0.0
Minocycline	8	≤0.5 to ≥32	–	–	4	≤0.5 to ≥32	–	–
Pip-taz	16	≤0.06 to ≥256	89.9	6.2	8	≤0.06 to 128	93.9	3.3
Tigecycline	2	0.015 to 8	80.7	2.6	2	0.03 to 4	80.3	1.1
*A. baumannii*	*N* = 1496				*N* = 270			
Amikacin	≥128	≤0.5 to ≥128	74.9	19.9	≥128	1 to ≥128	73.7	20.4
Cefepime	32	≤0.5 to ≥64	–	–	≥64	≤0.5 to ≥64	–	–
Ceftazidime (*N* = 1488)	≥64	≤1 to ≥64	–	–	32	≤1 to 32	–	–
Ceftriaxone	≥128	≤0.06 to ≥128	–	–	64	2 to 64	–	–
Levofloxacin	≥16	≤0.008 to ≥16	54.5	43.2	≥16	≤0.008 to ≥16	56.7	42.6
Meropenem^a^ (*N* = 1326)^b^	≥32	≤0.06 to ≥32	81.0	11.8	≥32	0.12 to ≥32	74.1	20
Minocycline	8	≤0.5 to ≥32	–	–	8	≤0.5 to ≥32	–	–
Pip-taz	≥256	≤0.06 to ≥256	–	–	≥256	≤0.06 to ≥256	–	–
Tigecycline	1	≤0.008 to 8	–	–	1	0.03 to 2	–	–
*A. baumannii* MDR	*N* = 93				*N* = 35			
Amikacin	≥128	32 to ≥128	0.0	100	≥128	32 to ≥128	0.0	100
Cefepime	≥64	8 to ≥64	–	–	≥64	8 to ≥64	–	–
Ceftazidime (*N* = 92)	≥64	≤1 to ≥64	–	–	32	2 to 32	–	–
Ceftriaxone	≥128	64 to ≥128	–	–	64	64 to 64	–	–
Levofloxacin	≥16	2 to ≥16	0.0	100	≥16	2 to ≥16	0.0	100
Meropenem (*N* = 92)^b^	≥32	16 to ≥32	0.0	100	≥32	16 to ≥32	0.0	100
Minocycline	16	≤0.5 to ≥32	–	–	16	≤0.5 to ≥32	–	–
Pip-taz	≥256	≤0.06 to ≥256	–	–	≥256	64 to ≥256	–	–
Tigecycline	4	0.12 to 4	–	–	2	0.25 to 2	–	–
*P. aeruginosa*	*N* = 2734				*N* = 738			
Amikacin	16	≤0.5 to ≥128	88.5	6.9	8	≤0.5 to ≥128	91.1	5.1
Cefepime	32	≤0.5 to ≥64	77.8	22.2	16	≤0.5 to ≥64	79.8	20.2
Ceftazidime (*N* = 2730)	32	≤1 to ≥64	77.2	22.8	32	≤1 to 32	80.2	19.8
Levofloxacin^c^	≥16	≤0.008 to ≥16	60.6	39.4	≥16	0.015 to ≥16	65.7	34.3
Meropenem (*N* = 2474)	8	≤0.06 to ≥32	74.6	8.7	16	≤0.06 to ≥32	75.2	10.0
Pip-taz^c^	128	≤0.06 to ≥256	74.4	25.6	128	≤0.06 to ≥256	78.7	21.3
Tigecycline	16	≤0.008 to ≥32	–	–	16	0.12 to 16	–	–
*P. aeruginosa* MDR	*N* = 271				*N* = 68			
Amikacin	≥128	1 to ≥128	31.4	58.3	≥128	2 to ≥128	38.2	50.0
Cefepime	≥64	2 to ≥64	14.0	86.0	≥64	4 to ≥64	8.8	91.2
Ceftazidime	≥64	2 to ≥64	21.4	78.6	32	4 to 32	23.5	76.5
Levofloxacin	≥16	0.5 to ≥16	1.5	98.5	≥16	2 to ≥16	0.0	100
Meropenem^a^ (*N* = 258)^b^	≥32	≤0.06 to ≥32	17.8	66.7	≥32	0.25 to ≥32	11.8	82.4
Pip-taz^c^	≥256	0.5 to ≥256	14.0	86.0	≥256	1 to ≥256	20.6	79.4
Tigecycline	≥32	1 to ≥32	–	–	16	2 to 16	–	–

– indicates no susceptibility breakpoints are available for this agent

^a^ indicates statistically significant decrease in susceptibility (p < 0.01) from 2004 to 2016

^b^ Meropenem was introduced to the testing panel in 2006, replacing imipenem; N values of activity against organisms collected from 2006 to 2016 are given

^c^ indicates statistically significant increase in susceptibility (*p* < 0.01) from 2004 to 2016

*Amox-clav*, amoxicillin-clavulanic acid, *BL*, β-lactamase, *ESBL*, extended-spectrum β-lactamase, *MDR*, multidrug-resistant, *MIC*, minimum inhibitory concentration, *MIC*_*90*_, minimum inhibitory concentration required to inhibit growth of 90% of isolates (mg/L), *Pip-taz*, piperacillin-tazobactam, *R*, resistant, S, susceptible

#### E. Coli

Isolates of *E. coli* (*N* = 3527) were highly susceptible to meropenem (99.9%), tigecycline (99.4%) and amikacin (98.1%) (Table 3). The susceptibility of *E. coli* isolates to piperacillin-tazobactam (89.6%) was relatively stable, but there was a decline in susceptibility to levofloxacin (92.1% in 2004 to 76.2% in 2016) and statistically significant declines in susceptibility to cefepime (97.0% in 2004 to 77.2% in 2016; *p* < 0.0001) and ceftriaxone (96.0% in 2004 to 78.2% in 2016; *p* < 0.0001).

The proportion of *E. coli* isolates identified as ESBL-positive *E. coli* between 2004 and 2016 (*N* = 489) was 13.9%. This is lower than the annual rates between 2013 (14.9%) and 2016 (19.8%), although these were stable (Table 2). Susceptibility of all ESBL-positive *E. coli* isolates was 99.2% to tigecycline, 92.6% to amikacin, and 100% to meropenem for the 472 isolates tested from 2006 onwards. Susceptibility of ESBL-positive *E. coli* to piperacillin-tazobactam (78.3%) was lower compared with all isolates of *E. coli* (89.6%), and only 37.8% of ESBL-positive *E. coli* isolates were susceptible to levofloxacin and 45.8% to amoxicillin-clavulanate; no isolates were susceptible to ceftriaxone and 3.9% were susceptible to cefepime.

#### H. Influenzae

*H. influenzae* isolates (*N* = 1786), including β-lactamase positive isolates (*N* = 410, 23.0%) collected between 2004 to 2016 (Table 3) were susceptible (> 91.0%) to agents in the T.E.S.T. panel with a breakpoint, with the exception of ampicillin, to which 75.4% of all *H. influenzae* isolates and 0.5% of β-lactamase positive isolates were susceptible.

#### Klebsiella spp

A total of 975 *K. oxytoca* isolates were collected during the study, and susceptibilities were highest to meropenem (*N* = 872, 99.8%), amikacin (98.9%) and tigecycline (95.8%). Over 80% of isolates were susceptible to cefepime, ceftriaxone, levofloxacin and piperacillin-tazobactam, and 79.8% of isolates were susceptible to amoxicillin-clavulanate.

Susceptibility of *K. pneumoniae* isolates collected between 2004 to 2016 (*N* = 2398) was highest to meropenem (*N* = 2186, 99.4%,), amikacin (96.5%) and tigecycline (87.4%) (Table 3). There was a significant (*p* < 0.0001) decline in susceptibilities to amoxicillin-clavulanate from 85.1% in 2004 to 46.2% in 2016, cefepime (95.5% in 2004 to 48.7% in 2016), ceftriaxone (91.0% in 2004 to 47.4% in 2016), levofloxacin (92.5% in 2004 to 66.7% in 2016) and meropenem (100% in 2004 to 92.3% in 2016).

The proportion of *K. pneumoniae* isolates identified as ESBL-positive between 2004 and 2016 (*N* = 622) was highest during 2016 (43.6%) (Table 2), an increase from 36.1% in 2013 and from 7.5% in 2004. Susceptibility was highest to meropenem (*N* = 603, 99.0%), amikacin (90.0%) and tigecycline (79.4%). Six *K. pneumoniae* isolates collected from one centre in 2016 were resistant to meropenem and these isolates were not ESBL-producers. Very few ESBL-positive isolates were susceptible to cefepime (5.0%) and ceftriaxone (1.3%) during the study, although susceptibility to levofloxacin improved to its highest level in 2016 (47.1%), and the susceptibility to piperacillin-tazobactam was 79.4% in 2016, a similar value compared with 80.0% susceptibility in 2004.

#### S. Marcescens

Between 2004 and 2016, 1345 isolates of *S. marcescens* were collected, and susceptibility was highest to meropenem (*N* = 1227, 99.1%), amikacin (97.3%) and cefepime (94.5%).

#### A. Baumannii

Few agents showed in vitro activity against *A. baumannii* isolates (*N* = 1496) (Table 3), with tigecycline and minocycline the two agents with relatively low MIC_90_ values (1 mg/L and 8 mg/L respectively); clinical breakpoints for these two agents are not available. Susceptibility to meropenem (*N* = 1326) was 81.0% and to amikacin 74.9%. There was a significant decrease (*p* < 0.0001) in the proportion of isolates that were susceptible to meropenem, from 84.8% in 2006 to 65.5% in 2016. None of the *A. baumannii* MDR isolates was susceptible to amikacin, levofloxacin (both *N* = 93) and meropenem (*N* = 92), the three agents with breakpoints. Antimicrobial activity of tigecycline against *A. baumannii* MDR isolates appeared reduced (MIC_90_ 4 mg/L) compared with all *A. baumannii* isolates. The proportion of *A. baumannii* MDR isolates increased from zero in 2004 to a high of 24.1% in 2016 (Table 2).

#### P. Aeruginosa

A total of 2734 *P. aeruginosa* isolates were collected during the study and susceptibility to antimicrobial agents was stable. Susceptibility was 88.5% to amikacin, whilst 77.8% of isolates were susceptible to cefepime, 77.2% to ceftazidime, 74.6% to meropenem and 74.4% to piperacillin-tazobactam. The proportion of *P. aeruginosa* isolates (*N* = 271) that were identified as MDR declined during the study from a high of 13.6% in 2013 to 4.0% in 2016, and susceptibility of these isolates was highest to amikacin (31.4%).

## Discussion

This report is an update to data previously presented by Cattoir and Dowzicky [17] for France, and includes data from isolates that were collected between 2004 and 2016. Data presented by Cattoir and Dowzicky that were based on isolates collected in France from 2004 to 2012 are included in the dataset we describe in this update.

The proportion of isolates identified as MRSA in our study was stable between 2004 and 2016, and averaged 27.7% compared with an average of 28.3% between 2004 and 2012 [17]. During the last four years of our study, there appeared to be a slight decline in MRSA rates from 28.0% in to 23.5%. Rates of MRSA in France were reported to be decreasing from 2003 to 2010 according to data from the RAISIN network published in 2013 by Carbonne et al. [2], and more recently the ECDC surveillance report identified an MRSA rate in France of 17.1% of invasive *S. aureus* isolates in 2013, 17.4% in 2014, 15.7% in 2015 and 13.8% in 2016 [3]. The use of control measures including isolation of patients with MRSA, the use of alcohol-based hand-rub, and screening of high-risk patients [9], have resulted in improved control of MRSA transmission in French hospitals [9, 22]. Consequently the proportion of *S. aureus* isolates identified as MRSA in France is showing a downward trend, and similar trends have been observed in Germany and the UK by the ECDC, which reported MRSA rates in 2016 of 10.3 and 6.7%, respectively [3]. Much higher MRSA rates have been reported in France’s neighbouring countries of Spain (25.8% in 2016) and Italy (33.6% in 2016) [3].

Susceptibilities of *S. aureus* isolates collected in our study were stable to tigecycline, vancomycin, linezolid and minocycline, including MRSA isolates, which showed susceptibility rates between 2013 and 2016 of 100% to tigecycline, vancomycin and linezolid and 95.3% to minocycline. The same values were reported by Cattoir and Dowzicky [17] for MRSA isolates collected between 2004 and 2012 (*N* = 631) for tigecycline, vancomycin and linezolid, with minocycline susceptibility similar at 93.5%. MRSA isolates collected in our study between 2013 to 2016 did not show any meaningful improvement in in vitro susceptibility to levofloxacin (17.5%) compared with 2004 to 2012 (13.2%) [17]. Beyond these favourable data, the spread of MRSA strains exhibiting a vancomycin MIC superior to 1 mg/L should be carefully monitored, according to their putative role in clinical therapeutic failure and additional associated resistance [23].

Susceptibility to vancomycin amongst Gram-positive isolates was 100% amongst *S. aureus, S. agalactiae* and *S. pneumoniae*, including resistant phenotypes. The proportion of *Enterococcus* spp. that were identified as vancomycin-resistant isolates from 2004 to 2012 by Cattoir and Dowzicky [17] was low (*E. faecalis* VRE 0.7%, *E. faecium* VRE 5.4%) and we report a similar observation after a further four years of study (2004 to 2016: *E. faecalis* VRE 0.8%; *E. faecium* VRE 5.6%).

There was a considerable reduction in the susceptibility of penicillin-resistant *S. pneumoniae* isolates to macrolides compared with all *S. pneumoniae* isolates in our study. However, susceptibility of penicillin-resistant *S. pneumoniae* was appreciably higher to erythromycin amongst isolates that were collected in our study between 2013 and 2016 (38.5%), compared with 19.4% susceptibility amongst isolates collected between 2004 and 2012 and reported by Cattoir and Dowzicky [17].

In our study, the proportion of ESBL-producers among *E. coli* (16.2%) between 2013 and 2016 represented a small increase compared with the 2004 to 2012 period reported by Cattoir and Dowzicky (12.0%) [17]. A study in France by Carbonne et al. on behalf of the RAISIN network reported a threefold increase in *E. coli* ESBL-producers identified from isolates collected from patients in participating healthcare facilities between 2003 and 2010 [2]. The increasing prevalence of ESBL-positive Enterobacteriaceae reported in healthcare settings is compounded by an increasingly frequent distribution in community settings. A recent study investigating risk factors of *E. coli* ST131 in children in the community found a doubling of ESBL-positive Enterobacteriaceae between 2010 and 2015 that was mainly attributed to the *E. coli* ST131 clonal group [24]. The spread of ESBL-positive Enterobacteriaceae in France appears to be due to CTX-M-type enzymes encoded in plasmids playing a major role, with three ESBLs (CTX-M-15, CTX-M-1, CTX-M-14) accounting for > 75% of isolates in a recent study of 200 clinical ESBL-positive samples collected from 18 French hospitals [4].

In our study, the in vitro susceptibility of tigecycline (99.5%), amikacin (98.4%) and meropenem (99.9%) observed against all *E. coli* isolates between 2013 and 2016 was retained among ESBL-positive isolates (99.4, 95.5 and 100%, respectively), and was similar to values for ESBL-positive *E. coli* reported by Cattoir and Dowzicky for the period 2004 to 2012 (tigecycline, 98.9%, amikacin 90.5%, meropenem 100%) [17]. Further comparison with the 2004 to 2012 dataset reveals an improvement in susceptibility of ESBL-positive *E. coli* isolates to amoxicillin-clavulanate (from 36.7% between 2004 to 2012 to 59.0% between 2013 to 2016) and to piperacillin-tazobactam (from 72.4% between 2004 to 2012 to 88.5% between 2013 and 2016). Susceptibility trends similar to those observed for *E. coli* isolates were observed amongst *K. pneumoniae* and ESBL-positive *K. pneumoniae* isolates for tigecycline, amikacin and meropenem. The sustained decline in susceptibility of *K. pneumoniae* to ceftriaxone during our study appears to be attributable to the increase in the proportion of ESBL-positive *K. pneumoniae* isolates that was observed as the study progressed, reaching its highest value of 43.6% in 2016. The high prevalence of *K. pneumoniae* isolates with antibiotic resistance has also been reported by the ECDC, which observed that 28.9% of *K. pneumonia* isolates from France in 2016 were resistant to third-generation cephalosporins, and the majority of these were ESBL-positive [3].

A recent study of infections caused by carbapenemases that were notified by local healthcare facilities to the French Institute for Public Health in France between 2004 and 2011 reported a sharp increase in annual reported episodes of CPE from three or less from 2004 to 2008, then six in 2009, 26 in 2010 and 13 in 2011 [5]. A total of 53 episodes were reported in all, and 42 were associated with cross-border transfers, suggesting that CPE were not endemic in France by 2011. Most CPE were mainly *K. pneumoniae* or *E. coli*, with the majority of carbapenemases identified as OXA-48 or a *K. pneumoniae* carbapenemase (KPC). A further study, by Dortet et al. [25], identified a more than twofold increase in Enterobacteriaceae isolates with decreased susceptibility to carbapenems that were received at the French Associated National Reference Centre from 2012 to 2014. The predominant carbapenemases identified in their study were OXA-48 variants. Despite apparent increases in the numbers of carbapenemases reported in France, the proportion of Enterobacteriaceae isolates with non-susceptibility to carbapenems would appear to remain very low; a rate of 0.63% was identified amongst 133,244 clinical isolates collected from 71 laboratories across France by Robert et al. [26], and 0.4% of *K. pneumoniae* isolates collected across France as part of the ECDC antimicrobial surveillance in Europe were identified as carbapenemase-resistant [3]. These findings are consistent with our study, in which almost all ESBL-positive isolates were susceptible to meropenem.

The proportion of *A. baumannii* isolates identified as MDR between 2004 and 2012 by Cattoir and Dowzicky was 4.7% [17], and although the proportion of MDR isolates increased considerably during the four further years of our study, increasing to 24.1% in 2016, the number of MDR isolates (*n* = 7) was low. A recent study in France of *A. baumannii* carbapenem non-susceptible isolates noted that the proportion of carbapenem non-susceptible strains amongst all *A. baumannii* isolates was low during 2001 and 2002, increased to 2.6% in 2003 and remained at ≤3.2% until 2009, when it increased to 5.0% of isolates or higher until the study concluded in 2011 [27]. The clinical threat presented by the increasing frequency of *A. baumannii* isolates that harbour carbapenemases is likely to be limited by the relatively low proportion of infections caused by *A. baumannii*, which were reported to account for just 0.02% of infections per 100 patients in French healthcare facilities in the 2012 French Point Prevalence Survey [28]. During our study, *A. baumannii* MDR isolates accounted for just 0.4% of all isolates collected between 2004 and 2016, suggesting that MDR *A. baumannii* is rare in France. Despite this, we report a notable fall in the in vitro susceptibility of MDR *A. baumannii* isolates to amikacin, levofloxacin and meropenem to the extent that none of the isolates was susceptible. Furthermore, the increase in the MIC_90_ value of tigecycline to 4 mg/L against MDR *A. baumannii* isolates from 1 mg/L against all *A. baumannii* isolates suggests a reduction in its antimicrobial activity, and underlines the paucity of effective antimicrobial agents that are available to physicians when treating infections caused by MDR *A. baumannii*.

Limitations of this study include a reduction in the number of centres in 2016 to four, which has the potential to magnify resistance rates should a single site experience a clonal outbreak or a resistant phenotype. There was one occurrence of this during 2016, when six ESBL-negative *K. pneumoniae* isolates from one centre were identified as resistant to meropenem. The source of one of these isolates was body fluids, and the remaining five were from faeces/stools. This outbreak was unlikely to significantly affect the antimicrobial susceptibility trends that we report, however there is the possibility of clonal outbreaks at a single site influencing the reported rates of resistant pathogens in our study. A further possible limitation might arise from the collection of isolates. The T.E.S.T. protocol specifies that each submitted isolate must be considered by the contributing centre to be the probable causative agent of an infection. Between 2004 and 2016, 36.4% (*N* = 772) of isolates from GI sources originated from faeces/stool (1.1% of the total number of isolates collected in the study), and it is conceivable that organisms identified from these isolates may not have been the probable causative agent of infection, a fact that has probably very slightly overestimated the resistance rates in Enterobacteriaceae. However, we would suggest that given the very low proportion of isolates obtained from this source, the overall trends we have observed in antimicrobial activity and rates of resistant phenotypes remain valid. Finally, although the report of global resistance rate is relevant, more accurate data according to the origin of the infection (i.e. community-associated or healthcare-associated) or the clinical context (e.g. bacteraemia, urinary tract infection, respiratory tract infection) should be of interest.

## Conclusions

During this study, nearly all (> 90.0%) Gram-positive isolates collected between 2004 and 2016 were susceptible in vitro to tigecycline, meropenem and linezolid, including MRSA and VRE phenotypes. Tigecycline and meropenem were also active in vitro against most Gram-negative isolates, including ESBL producers. The rates of MRSA and VRE we observed are stable, however there were notable increases in the rates of ESBL producers in *E. coli* and *K. pneumoniae*, accompanied by an increase in the proportion of *A. baumannii* isolates that were identified as MDR. These trends highlight the continued importance of surveillance studies for monitoring antimicrobial resistance and demonstrate the need for effective strategies to control the spread of resistant pathogens in hospital- and community-acquired infections in France.

## Abbreviations

cIAIs: Complicated intra-abdominal infections
CLSI: Clinical and Laboratory Standards Institute
CPE: Carbapenemase-producing Enterobacteriaceae
cSSTIs: Complicated skin and soft tissue infections
ECDC: European Centre for Disease Prevention and Control
ESBL: Extended-spectrum β-lactamase
EUCAST: European Committee on Antimicrobial Susceptibility Testing
GI: Gastrointestinal
IHMA: International Health Management Associates
KPC: *Klebsiella pneumonia* carbapenemase
MDR: Multidrug-resistant
MIC: Minimum inhibitory concentration
MIC_90_: MIC required to inhibit growth of 90% of isolates
MRSA: Methicillin-resistant *Staphylococcus aureus*
RAISIN: French national healthcare-associated infection early warning, investigation and surveillance network [Réseau d′alerte, d′investigation et de surveillance des infections nosocomiales]
T.E.S.T.: Tigecycline Evaluation and Surveillance Trial
VRE: Vancomycin-resistant enterococci
